# Impact and potential value of immunosenescence on solid gastrointestinal tumors

**DOI:** 10.3389/fimmu.2024.1375730

**Published:** 2024-06-28

**Authors:** Tianshuai Zhang, Rongbo Wen, Hao Fan, Yue Yu, Hang Jia, Zhiying Peng, Leqi Zhou, Guanyu Yu, Wei Zhang

**Affiliations:** Department of Colorectal Surgery, Shanghai Changhai Hospital, Naval Medical University, Shanghai, China

**Keywords:** gastrointestinal tumors, colorectal cancer, immunosenescence, tumor microenvironment, immunotherapy

## Abstract

Solid gastrointestinal tumors often respond poorly to immunotherapy for the complex tumor microenvironment (TME), which is exacerbated by immune system alterations. Immunosenescence is the process of increased diversification of immune genes due to aging and other factors, leading to a decrease in the recognition function of the immune system. This process involves immune organs, immune cells, and the senescence-associated secretory phenotype (SASP). The most fundamental change is DNA damage, resulting in TME remodeling. The main manifestations are worsening inflammation, increased immunosuppressive SASP production, decreased immune cell antitumor activity, and the accumulation of tumor-associated fibroblasts and myeloid-derived suppressor cells, making antitumor therapy less effective. Senotherapy strategies to remove senescent cells and block key senescence processes can have synergistic effects with other treatments. This review focuses on immunoenescence and its impact on the solid TME. We characterize the immunosenescent TME and discuss future directions for antitumor therapies targeting senescence.

## Background

Health problems caused by population aging are among the great challenges the world is facing today. Nearly half of the global disease burden (92 diseases (including 35 cancers), accounting for 51.3%, 95% uncertainty interval, 48.5–53.9) is considered age-related (1). These include colorectal cancer, a solid tumor of the gastrointestinal tract with the third-highest incidence and second-highest mortality rate globally (2). Cancer morbidity and mortality rates are the highest in individuals over 50 years of age, suggesting that aging may play a significant role in cancer development and progression (3). Studies on the mechanisms of aging and tumor development have shown that some hallmarks of aging (including genomic instability, epigenetic alterations, chronic inflammation, and dysbiosis) promote oncogenesis and progression, whereas others have shown antagonistic (telomere attrition and stem cell exhaustion) or ambivalent effects (disabled macroautophagy and cellular senescence) on tumors (4). Therefore, the effects of aging on tumors need to be specifically explored at the systemic, microenvironmental, and cellular levels.

The immune system constitutes the body’s defensive barrier by monitoring, protecting, and eliminating threats (5). However, the interaction between adaptive and innate immune cells can lead to chronic inflammation and increase the likelihood of cancer development, and different types of infiltrating immune cells can have opposite effects on tumor prognosis (6). In addition, immune system function decreases with age, known as immunosenescence, which increases the risk of cancer and is a key player in cancer development (7). It was Roy Walford who first elucidated the link between immunity and aging and coined the term “immunosenescence,” which refers to increased immunogenetic diversification due to aging, leading to a progressive decrease in the recognition function of the immune system (8, 9). Immune aging is not simply a one-way process that leads to dysfunction and other harmful effects, but a dynamic balance between adaptation and maladaptation (10).

Changes in the immune senescence process will further complicate the immune features of the tumor microenvironment (TME) and may therefore have diverse impacts on tumor development and immunotherapy. The TME is composed of multiple types of immune cells, cancer-associated fibroblasts, endothelial cells, pericytes, various tissue-resident cell types, and extracellular matrix (ECM) (11). The complexity of the TME lies in the fact that immune cells are recruited to and infiltrate the TME through the action of cytokines and chemokines secreted from cancer cells to play an antitumor role, but simultaneously produce additional features of the TME that facilitate immunosuppression and limit antitumor immune responses (12–14). Particularly in colorectal cancer, slight alterations in the TME will trigger complex immunotherapy changes (15). Increasing evidence suggests that both innate and adaptive immune cells in the TME have a facilitative effect on tumor progression, while crosstalk with cancer cells enhances the recruitment of suppressive immune cells, including myeloid-derived suppressor cells (MDSCs) and tumor-associated macrophages (16–18). In addition, a decrease in antitumor immune cell infiltration and function, together with the accumulation of immunosuppressive cells and upregulation of ligands that bind to inhibitory receptors on immune cells, may contribute to immune escape and consequently lead to poor immunotherapy results (19, 20).

Although remarkable research advancements have been made for both immunosenescence and the TME in the past decades, the impact of their interaction on different constituents and tumor progression remains to be further explored. This review focuses on the process of immunosenescence and the role of TME regulation. In addition, we discuss the impact of immunosenescence on tumor progression and immunotherapy. Finally, we describe future directions for limiting tumor progression by intervening in immunosenescence.

## The process of immunosenescence

**Figure 1:**
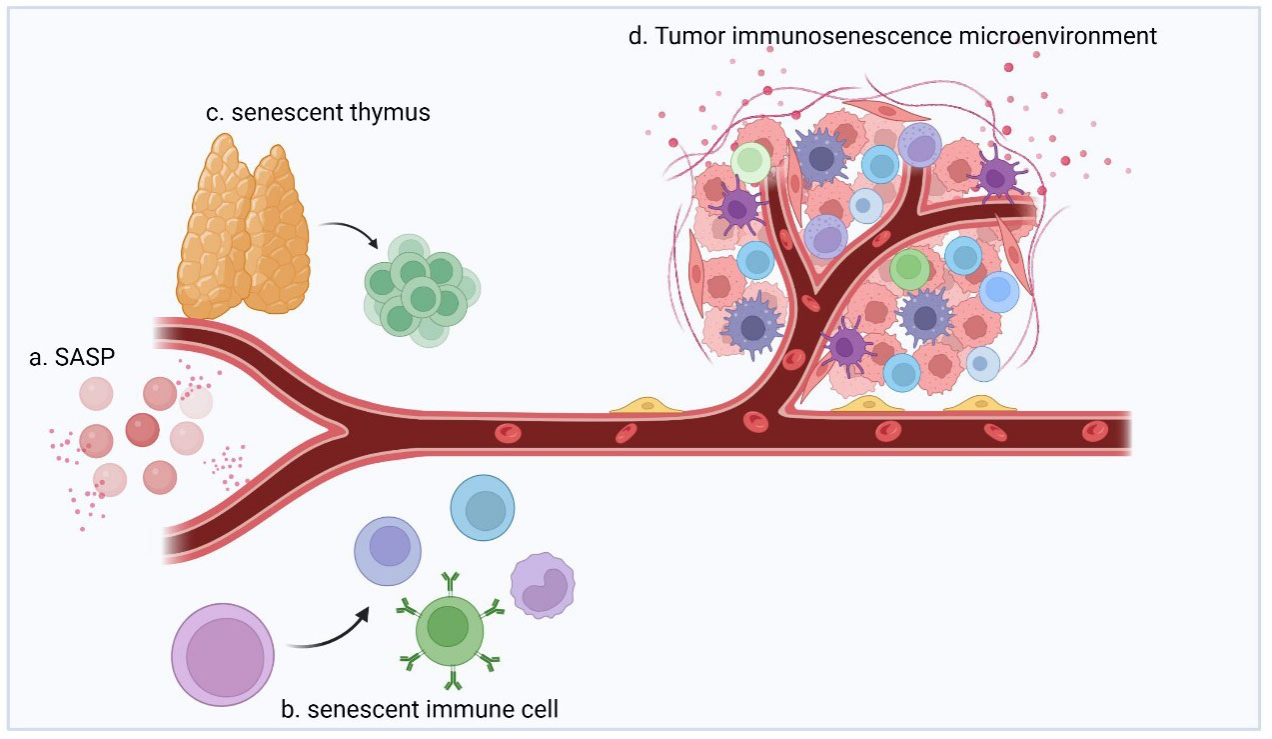
The immunosenescence process involves immune organs, immune cells, and circulating factors (chemokines, cytokines, and other soluble molecules) as three distinct but interrelated components that undergo changes during aging, with corresponding effects. **(A)** During aging, senescent cells secrete signaling and action molecules such as inflammatory, extracellular modifying, and growth factors, collectively known as SASP, which are key factors in facilitating and mediating immunosenescence. **(B)** Immune cells produced by senescent hematopoietic stem cells interact with SASPs and are characterized by increased numbers, increased secretion of inflammatory factors, decreased self-renewal capacity, diminished homing effects, and decreased energy metabolism. **(C)** During aging, thymus cells are gradually replaced by adipocytes, which results in a decrease in the proportion of undifferentiated T cells produced by the thymus (e.g., naïve T cells). **(D)** Multiple factors act together to shape the immunosenescent TME and exhibit strong immunosuppressive effects.

The process of immune aging involves three distinct but interrelated components, i.e., the immune organs, immune cells, and circulating factors (chemokines, cytokines, and other soluble molecules), which change during aging and produce corresponding effects (**Figure 1**) (21). Immune system aging ultimately results in increased incidence of infectious diseases and mortality, reduced responsiveness to vaccines, accelerated aging of other organs, and increased risk of tumors (22–25) Immunosenescence is a complex and well-integrated process.

The aging of immune organs is the most noticeable change. For example, thymus function degenerates in nearly all species. Thymic involution begins in childhood and reaches its peak in adolescence. While excessive energy use is reduced in this process, age-related degeneration is detrimental to the organism (26, 27). During this process, thymus cells are gradually replaced by adipocytes, which results in a decrease in the proportion of undifferentiated T cells produced by the thymus (e.g., naïve T cells) and an increase in that of terminally differentiated cells (e.g., memory or depleted phenotypic T cells) (28). Such changes are also observed in neonates with early thyme resection, suggesting that they are a sign of immune deficiency (29). In conclusion, thymic degeneration is associated with the age-related immune decline and makes one prone to age-related diseases.

The key factors in promoting and mediating immunosenescence are alterations in circulating factors (chemokines, cytokines, and other soluble molecules). Immunosenescence causes the body to gradually enter an age-related pro-inflammatory state, while simultaneously, the body exerts anti-inflammatory effects through low-level, sterile chronic inflammation to adapt and remodel the immune system (30). During this process, senescent cells secrete inflammatory, extracellular modifying, and growth factors as signaling and acting molecules collectively referred to as the senescence-associated secretory phenotype (SASP) (31).

**Figure 2:**
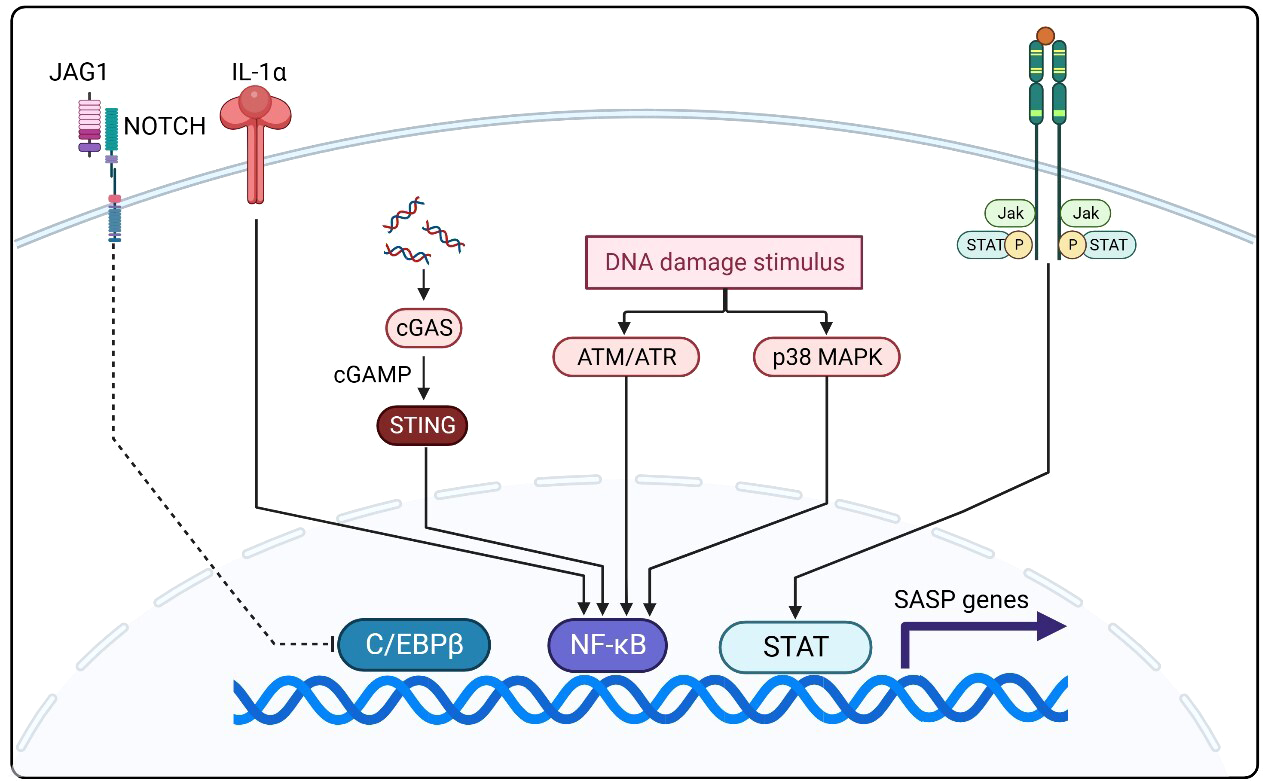
NF-κB activation is closely related to SASP regulation and activation. The p38 MAPK, ATM/ATR, and cGAS-STING pathways and aberrant IL-1α activation mediate SASP production by NF-κB. The NOTCH-JAG1 pathway can synergize with NF-κB to activate SASP production by inhibiting C/EBPβ. In recent years, JAK/STAT pathway activation by signaling molecules including phospholipase A2 receptor 1 (PLA2R1), TNF-production by iN-γ has been shown to also induce SASP production.

The SASP is expressed upon exposure to excessive stresses, such as repetitive cell division, oxidative stress, mitochondrial degradation, oncogene expression, and other stresses that cause DNA damage (**Figure 2**) (32). As an inflammatory response, the regulation of SASP is strongly associated with nuclear factor kappa-light-chain-enhancer of activated B cells (NF-κB) activation. As a classical DNA damage response pathway, the p38 MAPK pathway is activated by oxidative stress and DNA damage and regulates NF-κB through the p16^INK4A^, p53, and DNA damage checkpoint kinase CHK1/CHK2 mechanisms, which in turn produce the SASP (33–37). Another DNA damage response-related pathway, the ATM/ATR pathway, is thought to mediate NF-κB action via the key molecule GATA4 to produce the SASP (38). In addition, the downregulation of DNase (DNase2/TREX1) expression in senescent cells leads to the accumulation of DNA in the cytoplasm, which in turn leads to abnormal cGAS-STING pathway activation and SASP production through IFN-mediated NF-κB activation (39). Another pathway validated to produce SASP via NF activation is regulated by IL-1α, which phosphorylates IRAK1 via IRAK4 after binding to the IL-1 receptor and eventually activates NF-κB (40). Another signaling molecule that can regulate SASP is NOTCH1, which acts synergistically with NF-κB by activating the NOTCH-JAG1 pathway to produce TGF-β to induce aging while inhibiting C/EBPβ (41). In recent years, JAK/STAT pathway activation by signaling molecules including phospholipase A2 receptor 1 (PLA2R1), tumor necrosis factor (TNF)-α, and interferon (IFN)-γ has been shown to also induce SASP production (42, 43). The SASP generated through multiple pathways will profoundly impact immune cell function and ultimately restructure the TME.

Alterations in immune cells are the most complex part of immune aging and produce direct effects. Such alterations are mainly due to two aspects: on the one hand, as mentioned above, the SASP plays a regulatory role in immune cell senescence, and on the other hand, hematopoietic stem cell (HSC) senescence is considered to be the basis of immunosenescence (44). Inflammation is a major factor in HSC aging, as inflammatory factors such as IL-1, IFNα/γ, and TNF-α drive HSC aging (45–47). Aging HSCs and immune cells differentiated from HSCs are increased in numbers and show increased inflammatory factor secretion, reduced self-renewal capacity, diminished homing effects, and reduced energy metabolism (44). Aging immune cells interact with soluble factors, including the SASP, in the TME to influence tumor progression and therapeutic efficacy, reflecting the impact of immunosenescence on cancer.

## Impact of immunosenescence on TME alterations and tumor progression

The TME consists of various components that can be classified into a non-cancerous cellular fraction, including fibroblasts, neurons, adipocytes, and immune cells (adaptive and innate), and a non-cellular fraction, including ECM, chemokines, growth factors, cytokines, and vesicles (48). According to the characteristics of each component, the TME can also be subdivided into tumor immune microenvironment, tumor biophysical microenvironment, tumor microbe microenvironment, etc. (49–51) We propose the term “immunosenescence microenvironment” as a new TME component to reflect the impact of senescent immune-related cells and signaling molecules in the TME on tumor development (**Table 1**). By analyzing the individual components of the immunosenescent TME, we can more clearly the delineate the role of immunosenescence on tumor development.

**Table 1:** Changes in components of the tumor immunosenescence microenvironment.

Components		Specific changes and impacts	Ref.
SASP	IL-6	Drive tumor cell proliferation, invasion, and metastasis; reduce tumor antigen expression as well as genotoxic stress; promote neoangiogenesis.	(50–54)
	IL-8	Drive tumor cell proliferation, invasion, and metastasis.	(55–57)
	IL-1α/β	Induce the expansion of myeloid-derived suppressor cells (MDSC); decrease the activity of NK cells and CD8+ T cells; increase inhibitory anti-tumor immune cells such as Treg cells and M2 macrophages.	(58–60)
	CCL5	Recruit immunosuppressive lymphocytes such as Treg cells.	(61)
	CXCL1	Recruit MDSCs; reduce CD8+ T cells.	(61–64)
	CXCL2	Recruit MDSCs; reduce CD8+ T cells.	(61–64)
	CXCL5	Recruit MDSCs.	(61, 64–66)
	CXCL12	Attenuates T-cell infiltration and tumor cell killing ability; increases tumor angiogenesis and immune resistance.	(61, 67–69)
	TNF-α	Mediate cell death.	(70)
	VEGF	Promote tumor angiogenesis.	(71)
	GM-CSF	Induce immune cell depletion	(72)
T cell		Competitive grape depletion with Treg cells.	(73, 74)
		Tumor-derived cyclic adenosine monophosphate (cAMP) and some genotoxins of pathogenic bacteria can induce senescence of T cells through DNA damage.	(75–77)
		DNA damage produced by this process is mainly regulated by the MAPK and STAT pathways.	(78–80)
		TNFα and proteases are the main components of SASP secreted by T cells.	(81)
		Down-regulation of CD27, CD28, and the up-regulation of CD57.	(82–85)
		Decreased production of perforin, which reduces cytolysis and tumor cell killing.	(86)
B cell		Deterioration of the inflammatory state of the tumor microenvironment by a class of B cells called age-associated B cells (ABCs).	(87, 88)
		IgD CD27 double-negative B cells (DN cells) accumulate in areas of chronic inflammation and exacerbate the inflammatory microenvironment by producing pro-inflammatory factors.	(89, 90)
NK cell		The proportion of CD56dim increases while the proportion of CD56bright decreases.	(91)
		Cytotoxicity was attenuated by decreased perforin production and decreased degranulation.	(92, 93)
Other cells	DCs	The endocytosis, and presentation of antigens by dendritic cells are diminished, while more pro-inflammatory cytokines are secreted.	(94)
		Reduced ability to activate T cells.	(95, 96)
	MDSCs	Produce inflammatory molecules such as IL-10 and TGF-β with inhibitory antigen presentation or immunosuppressive effects.	(97–99)
		Enhancement of oxidative stress in the microenvironment by generation of reactive oxygen species and inhibition of immune checkpoint protein-mediated contact between T cells and tumor cells	(100–102)

### SASP

A large number of SASP signaling molecules originate from the ECM, which plays a microenvironmental regulatory role in the immunosenescent TME, and these molecules determine the overall state of the TME (52). The SASP, derived from senescent cells, plays an important regulatory role in antitumor immunity in the ECM. Its effects are generally mediated by the paracrine way and have both positive and negative effects on tumor progression (53).

Various factors of the interleukin (IL) family involved in the SASP, such as IL-6, IL-8, and IL1α/β, function in microenvironment regulation. The IL-6/JAK/STAT3 signaling pathway drives tumor cell proliferation, invasion and metastasis and suppresses anti-tumor immune responses by reducing tumor antigen expression and decreasing responses to genotoxicity (54–57, 103). IL-6 as well as IL-8 can enhance tumor metastasis by promoting neoangiogenesis (58–61). IL-1β mediates immunosuppression by NLRP3 by inducing the expansion of MDSCs, leading to a decrease in the activity of natural killer (NK) cells and CD8+ T cells and an increase in the number of inhibitory antitumor immune cells, such as regulatory T (Treg) cells and M2 macrophages, in the TME (62, 63). Similarly, IL-1α/β secreted by tumor cells also induces fibroblasts to release pro-tumorigenic chemokines including CXCL9 and CXCL10 (65).

Chemokines involved in the SASP are another important type of regulatory molecules in the TME. CCL5, CXCL1, CXCL2, CXCL5, and CXCL12 are chemokines produced by senescent cells that have opposite effects on tumor development (66). Chemokines such as CCL5 recruit antitumor immune cells to enhance antitumor immunity while recruiting immunosuppressive lymphocytes such as Treg cells, leading to tumor immune escape (64, 67, 68). On the contrary, CXCL5, CXCL1 and CXCL2 have a tumor-promoting effect because they recruit MDSCs, which can play an immunosuppressive role by suppressing the immune function of lymphocytes through the secretion of Arg-1 and iNOS, and CXCL1 and CXCL2 also reduce the number of CD8+ T cells (69–72, 75, 76). CXCL12 attenuates T-cell infiltration and tumor cell-killing ability and increases tumor angiogenesis and immune resistance via CXCR4/CXCL12 (73, 74, 77).

Other important modulations of tumor progression by the SASP include TNF-α-mediated cell death, vascular endothelial growth factor-promoted tumor angiogenesis, and granulocyte macrophage colony-stimulating factor-induced immune-cell depletion, which inhibits antitumor immunity and promotes tumor progression (78, 79, 104).

### T cells

Tumor cells and Treg cells are thought to induce T-cell senescence directly, and some senescent cells secrete SASPs that may have a consistent effect. Tumor-derived cyclic adenosine monophosphate can cause DNA damage and senescence in both CD4+ T cells and CD8+ T cells and immunoglobulin-like transcript 4 and its derivative PIR-B induce T cell senescence by increasing the fatty acid synthesis and lipid accumulation in tumor cells via MAPK ERK1/2 signaling (80). In CD4^+^ T cells, AMPK can trigger p38 phosphorylation via the scaffolding protein TAB1, which in turn activates the MAPK signaling pathway to induce senescence (81). While in CD8+ T cells, activation of the p38 MAPK pathway leads to the secretion of SASP (82).

Treg cells also play an essential role in inducing T-cell senescence. Treg cells have a selective metabolic profile that accelerates glucose depletion compared to effector T cells and suppresses responding T cells and induces senescence through cross-talk (83). This is because metabolic competition controls DNA damage in effector T cells through ERK1/2 and p38 signaling in cooperation with STAT1 and STAT3, leading to senescence and functional changes that are molecularly distinct from energy and exhaustion (105). Some genotoxins from pathogenic bacteria can also induce CD4^+^ T-cell senescence through DNA damage, suggesting that the gastrointestinal microbiota may complicate the tumor immunosenescence microenvironment (84).

CD8^+^ T cells are key immune cells that exert tumor-cell killing; therefore, their senescence significantly affects antitumor capacity. Changes in surface costimulatory molecules such as CD27, CD28, and CD57 reduce the tumor-associated antigen recognition ability of CD8^+^ T cells, resulting in decreased antitumor activity of CD8^+^ T cells (85, 86, 106). Further, decreased perforin production by senescent CD8^+^ T cells reduces cytolysis and decreases their tumor cell-killing function (107). However, a recent study came to the opposite conclusion, suggesting that the effect of aging on the ability of CD8^+^ T cells to kill tumor cells needs to be further explored (108). Research on T-cell senescence is limited, but some hallmarks of T-cell senescence have been identified (109). The DNA damage produced during the process is mainly regulated by the MAPK and STAT pathways (81, 87, 110). TNFα and proteases are the main SASP components secreted by senescent T cells (82). Changes in T-cell surface proteins, including the downregulation of CD27 and CD28 and the upregulation of CD57, are one of the hallmarks (88, 89). Further, senescent T cells enter cell-cycle arrest after T-cell receptor stimulation (83, 90, 91, 111).

### B cells

B cells in TME can produce antibodies that bind to tumor-associated antigens and exert antitumor effects of antigen presentation (92). Their senescence arises predominantly from a decrease in B-cell differentiation and maturation in the bone marrow due to HSC senescence, as well as the reorganization of peripheral B-cell subsets (112, 113).

The impact of senescent B cells on TME is reflected not only in a decrease in antigen-presenting capacity but also in the pro-inflammatory effects derived from a class of B cells known as “age-associated B cells”, which are thought to be associated with TNFα secretion (93, 94, 114). IgD CD27 double-negative B cells, which play an immunosuppressive role, are another type of B cell that expands in aging populations (95). These cells are more likely to aggregate in areas of chronic inflammation due to surface expression of CCR6 and CCR7 after senescence and exacerbate the inflammatory microenvironment through pro-inflammatory factor production, worsening the immunosuppressive function of the TME (96, 97).

### NK cells

NK cells are a key innate immune component of the TME that exerts antitumor immunity. These cells are more environmentally sensitive, as evidenced by the fact that passive transfer to environments of different age states can have a significant effect on cytotoxicity (115).

In senescent NK cells, the proportion of CD56^dim^ increases, whereas that of CD56^bright^ declines, which in turn leads to diminished immunocyte function (116). Senescent NK cells appear to enter a silent phase, as cytotoxicity after senescence is attenuated by reduced perforin production and decreased degranulation, and even the production of cytokines such as IFN-γ, MIP-1α, and IL-8 after stimulation is lower than that in nonsenescent NK cells (117–120).

### Other cells

Dendritic cells are important antigen-presenting cells that play an important coordinating role in the immune response (121). However, as a result of immune senescence, the endocytosis and presentation of antigens by dendritic cells are diminished, while more pro-inflammatory cytokines are secreted (98). In addition, the ability of dendritic cells to activate T cells is reduced by senescence (99, 100).

MDSCs are a class of immunosuppressive cells recruited by the chronic inflammatory tumor immunosenescence microenvironment (101). MDSCs can inhibit the anti-tumor function of T cells and NK cells by expressing immune checkpoint molecules such as PD-L1 (102, 122, 123). In addition, MDSCs can affect the normal amino acid metabolism of T cells by depriving them of cysteine. This process affects the utilization of tryptophan by T cells and produces the immunosuppressive metabolite l-kynurenine, which ultimately induces T cell loss of function and promotes Treg cell differentiation (124, 125).

MDSCs drive immunosenescence and structure the immunosuppressive microenvironment, which are correlated with their multiple immunosuppressive functions (126). Upon the activation of MDSC amplification due to chronic inflammation caused by tumors and aging, certain chemokines, such as CCL2, CXCL1, and CXCR2, can recruit them to the TME, and this process can be enhanced by the complex gastrointestinal bacterial environment (127–132). Inflammatory molecules in the TME activate the immunosuppressive function of MDSCs mainly via JAK-STAT and NF-κB signaling, causing MDSCs to produce inflammatory molecules such as IL-10 and TGF-β that have antigen presentation-inhibitory or immunosuppressive effects (101, 133, 134). In addition, the immunosuppressive effects of MDSCs are reflected in the enhanced oxidative stress state in the microenvironment via the active generation of reactive oxygen species and the inhibition of immune checkpoint protein-mediated contact between T cells and tumor cells (135–137).

Other cells such as macrophages and neutrophils also exhibit immunosuppressive effects in the immunosenescent TMEby exacerbating chronic inflammation and increasing pro-tumorigenic M2-type macrophages (138). These molecular and cellular changes in the TME in the context of immune senescence promote tumor progression to a certain extent, and more importantly, influence antitumor therapy.

## Immunosenescence and tumor therapy

The relationship between immunosenescence and antitumor therapy reflects the fact that there always are two sides to the same coin. On the one hand, for gastrointestinal solid tumors such as colorectal tumors, radiotherapy, chemotherapy, and immunotherapy are important antitumor treatments besides surgery; however, these treatments induce immune senescence, termed “treatment-induced immune senescence.”

Radiotherapy and chemotherapy can cause cancer-associated fibroblasts to expand in the TME and exacerbate the inflammatory state, leading to immune senescence (139). In addition, radiotherapy and chemotherapy can accelerate cellular senescence by directly causing DNA damage (140–142). Immunotherapies such as immune checkpoint inhibitors can also induce senescence in TME components by inducing increased production of senescence-related cytokines (143).

## Senotherapy strategies targeting immunosenescence

On the other hand, senescence can be exploited as a new target in antitumor therapies (**Table 2**) (144, 145). This is explained by the fact that senescent cells continue to secrete SASP and lead to the presence of a pro-tumoral tumor microenvironment. Thus, senotherapy refers to the rational use of treatments that target senescent cells to fight tumors (146).

**Table 2:** Therapeutic strategies to counteract the immunosenescent microenvironment of tumors.

Treatment strategy		Key points	Ref.
Senotherapy	Removal of senescent tumor cells.	Inhibit the senescent cell anti-apoptotic protein BCL family.	(132, 133)
	Extensively removes senescent cells.	Inhibit the pro-survival network that is upregulated in senescent cells	(134)
	Removal of senescent cells using cellular engineering.	Using CAR-T cells to target the characteristic protein urokinase-type fibrinogen activator receptor (uPAR) on the surface of senescent cells.	(137)
	Clearance of tumor cells after active induction of senescence.	Selective induction of TP53-mutated cancer cell senescence using the DNA replication kinase CDC7 allows subsequent inhibitors of mTOR signaling to sustainably suppress and kill tumor cells.	(138)
	Mitigate the negative effects of SASP.	Inhibit SASP-producing pathways such as the previously mentioned p38 MAPK, NF-κB, and JAK/STAT	(139, 140)
Enhancing anti-tumor immune efficacy in the tumor immunosenescence microenvironment		Increased CD8^+^ T-cell infiltration using photochemotherapy.	(141)
		Δ133p53α TCR-T cell enhances fitness and effector functions of senescent T cells by modulation of p53 isoforms	(142)

One senotherapeutic strategy is the removal of senescent cells by using anti-aging cell drugs that complement other antitumor therapies by mitigating the negative effects of treatment-induced senescence. Some drugs are used after senescence-inducing cancer therapies to target senescent tumor cells for clearance (147). BCL family inhibitors (e.g., ABT-737 and ABT-263) are representative of this class of drugs; they scavenge senescent cells by inhibiting the anti-apoptotic BCL protein family (148, 149). The tyrosine kinase inhibitor dasatinib combined with quercetin selectively kills senescent cells by inhibiting the pro-survival network that is upregulated in senescent cells (150). In addition, there are immunotherapeutic drugs and antibody-drug combinations that can similarly remove senescent cells and synergize with senescence-inducing antitumor treatments (151, 152). Engineered CAR-T cells targeting urokinase-type plasminogen activator receptor, a characteristic protein on the surface of senescent cells, can be used as a therapeutic modality to remove senescent cancer cells (153). Another senotherapeutic strategy is to induce senescence of tumor cells and then target them for elimination. The key to this strategy is to find corresponding drugs that can be targeted to induce tumor-cell senescence. For example, the DNA replication kinase CDC7 can selectively induce senescence in TP53-mutated hepatocellular carcinoma cells, which can subsequently be killed by mTOR signaling inhibitors (154). Mitigating the negative effects of the SASP is another senotherapeutic strategy. This approach is mainly based on the inhibition of SASP-producing pathways, such as the above-mentioned p38 MAPK, NF-κB, and JAK/STAT pathways (155, 156).

Other therapeutic approaches, such as the use of engineered tumor-targeting TCR-T cells or of photochemotherapy to increase immune cell infiltration, have been successful in countering the immunosenescent TME by enhancing antitumor immune efficacy (157, 158).These diverse therapeutic approaches combined can exert a more significant antitumor effect by targeting the immunosenescent environment from different angles.

## Emerging biomarkers of immunosenescence in gastrointestinal tumors

Although the molecular biology of immunosenescence has been explored and therapeutic strategies for tumors have been optimized based on its action mechanisms, the timely identification of immunosenescent phenotypes is more clinically relevant, which provides an opportunity for early intervention (159, 160).

Since aging occurs in the immune system, accordingly, the type of biomarker that most readily comes to mind is the senescent phenotype of immune cells. Of these, both CD8^+^ TEMRA (CD45RA^+^CCR7^-^CD28^-^CD27^-^) and CD4^+^ TEMRA are markers of immunosenescence, with increased proportions and absolute numbers in colorectal cancer patients, reflecting low value-added potential and anti-apoptotic properties (161, 162). However, these markers are cell surface receptors and must be assayed using flow cytometry, which requires fresh blood samples. In contrast, the soluble form of immunosenescence markers are more stable and can be measured from stored serum, making them promising candidates as soluble markers of immunosenescence. However, these markers are less specific, as they can also be detected during acute inflammation. The soluble markers sCD163, sCD28 and sCTLA-4 have great potential for application as biomarkers of immunosenescence (163–165). These soluble markers can be detected using ELISA methods, but further studies are needed to compare the diagnostic performance of these markers with the gold standard (cell surface receptor) assay. In addition, some mutations in genes associated with immunosenescence such as PIK3CA, TP53, NF-κB, AMPK, mTOR, and P53 may also serve as biomarkers of the aging process (166–169).

## Conclusions

Immunosenescence, as a feature of this stage, increases the risk of infectious diseases and tumors while decreasing antitumor immune functions. This is because immune senescence results in changes in immune organs, immune cells, and the SASP, which all interact with each other. Such changes significantly impact solid tumors of the gastrointestinal tract, such as colorectal cancer, which have a complex TME, and ultimately lead to the formation of an immunosuppressive tumor immunosenescence microenvironment. In such an environment, immunosuppression is manifested in multiple aspects, including immunosuppressive cell recruitment, increased secretion of inhibitory cytokines, and diminished antitumor immune cell function. These changes allow the tumor to develop and deteriorate, and increase its tendency to invade, and affect antitumor therapy, which in itself induces immune senescence and induces immunosuppressive changes. Therefore, senotherapy, a new therapy targeting immune senescence, has been developed on the basis of various antitumor therapies to remove senescent tumor cells and restore the antitumor capacity of immune cells from a different perspective. However, more in-depth studies on the tumor immune senescence microenvironment need to be conducted to paint a more complete picture of immunosuppression and explore the mechanisms by which immunosenescence attenuates antitumor immunity. This will enable the development of antitumor drugs and different therapeutic strategies for aging characteristics. However, the therapeutic effects remain to be verified in long-term experiments.

## Author contributions

TZ: Conceptualization, Investigation, Writing – original draft. RW: Conceptualization, Investigation, Methodology, Writing – original draft. HF: Conceptualization, Investigation, Writing – original draft. YY: Conceptualization, Formal Analysis, Writing – original draft. HJ: Data curation, Writing – original draft. ZP: Data curation, Writing – original draft. LZ: Investigation, Supervision, Writing – review & editing. GY: Funding acquisition, Supervision, Writing – review & editing. WZ: Funding acquisition, Project administration, Supervision, Writing – review & editing.
